# Conical shell X-ray beam tomosynthesis and micro-computed tomography for microarchitectural characterisation

**DOI:** 10.1038/s41598-023-48851-6

**Published:** 2023-12-06

**Authors:** Emily L. Arnold, Farid Elarnaut, David Downes, J. Paul O. Evans, Charlene Greenwood, Keith D. Rogers

**Affiliations:** 1 Cranfield Forensic Institute, Cranfield University, Shrivenham, SN6 8LA Wiltshire UK; 2 Imaging Science Group, Nottingham Trent University, Rosalind Franklin Building, Nottingham, NG11 8NS UK; 3 School of Chemical and Physical Sciences, Keele University, Keele, ST5 5BJ Staffordshire UK

**Keywords:** Biomedical engineering, Bone

## Abstract

Bone quality is commonly used to diagnose bone diseases such as osteoporosis, with many studies focusing on microarchitecture for fracture prediction. In this study a bovine distal femur was imaged using both micro-computed tomography (µCT) and tomosynthesis using focal construct geometry (FCG) for comparison of microarchitectural parameters. Six regions of interest (ROIs) were compared between the two imaging modalities, with both global and adaptive methods used to binarize the images. FCG images were downsampled to the same pixel size as the µCT images. Bone morphometrics were determined using BoneJ, for each imaging modality, binarization technique and ROI. Bone area/total area was found to have few significant differences between FCG and µCT (p < 0.05 for two of six ROIs). Fractal Dimension had only one significant difference (p < 0.05 for one of six ROIs) between µCT and downsampled FCG (where pixel size was equalized). Trabecular thickness and trabecular spacing were observed to follow trends as observed for the corresponding µCT images, although many absolute values were significantly different (p < 0.05 for between one and six ROIs depending on image types used). This study demonstrates the utility of tomosynthesis for measurement of microarchitectural morphometrics.

## Introduction

Osteoporosis is a sweeping condition which currently affects over 3.7 million people in the UK^1^, and is estimated that the treatment and prevention will cost £5.5 billion annually by 2025^2^. Currently, dual energy X-ray absorptiometry (DXA) is used as the ‘gold standard’ for diagnosis of osteoporosis^3^ by measuring bone mineral density (BMD) at the lumbar spine and hip. DXA is often used in conjunction with additional tools such as FRAX®^4^ for improved fracture prediction. Bone strength is thought to be a better predictor for fracture than BMD alone, encompassing both microarchitectural and physicochemical ‘bone quality’^5–7^. In an effort to better quantify bone quality, through several different imaging techniques, a number of studies have shown that microarchitectural parameters change significantly in diseased bone compared to healthy bone, including trabecular thickness, trabecular separation, and fractal dimension^8–12^.

Recently studies have investigated the use of high resolution peripheral quantitative computed tomography (HR-pQCT) for fracture prediction^13,14^, cone beam CT (CBCT)^15^, and magnetic resonance imaging (MRI)^16^, often compared to micro-CT (µCT). Currently, HR-pQCT can achieve the highest resolution, at an isotropic resolution of either 61 µm or 82 µm, while in practice it achieves a spatial resolution of 100 µm or 142 µm^14^. However, a considerable limitation of HR-pQCT remains that it can only be used to examine peripheral sites. Relatively recent studies have used MRI to image trabecular bone and achieved an in-plane resolution of 156 µm with a slice thickness of 0.5 mm at the distal radius^17^, and an in-plane resolution of 234 µm with a slice thickness of 1.5 mm at the hip^16^. In comparison, CBCT can achieve a voxel size between 75 µm and 500 µm^15^. Additionally, opportunistic screening using clinical CT is a relatively common topic within literature^18^, as is the use of more traditional digital tomosynthesis for fracture prediction^19^.

Focal construct geometry (FCG) is an alternative X-ray beam geometry which uses a conical shell X-ray beam, designed to reduce exposure time for collection. It also provides unique access to the coherent scatter signals, thus providing crystallographic detail of the inspected material. FCG has been applied to X-ray absorption imaging^20,21^ and X-ray diffraction (XRD) in energy dispersive^22^ and angular dispersive modes^23–25^, notably on bone^22,26^. Recently this approach has been expanded to benefit from sporadic collection to further reduce dose and scanning time^21^. Additionally, a high-resolution technique using interleaved sampling has been applied to decrease pixel size in the *xy* plane^27^ by an order of magnitude.

To evaluate the application of absorption FCG imaging within a clinical setting, and further the analytical methodology introduced previously^27^, this work compares FCG to µCT imaging quantified through measurement of trabecular microarchitecture morphometrics in a distal bovine femur. To evaluate the accuracy of microarchitectural parameters measured from absorption FCG imaging, six regions of interest (ROIs) were selected, and four parameters were measured for each imaging modality. This analysis demonstrates the ability of FCG to measure microarchitectural properties, potentially useful for clinical diagnostics in the future.

## Methods

### Sample

A distal bovine femur was sourced from a local butcher (18–24 months), approximately 115 mm × 144 mm × 99 mm.

### µCT

The bone sample was scanned using a Nikon CT H225 (X-Tek Systems LTd, Tring, Hertfordshire, UK) cone beam µ-CT scanner operated at 125 kV and 101 µA, with a voxel size of 125 µm. Noise correction and beam hardening corrections were applied.

### FCG

**Figure 1:**
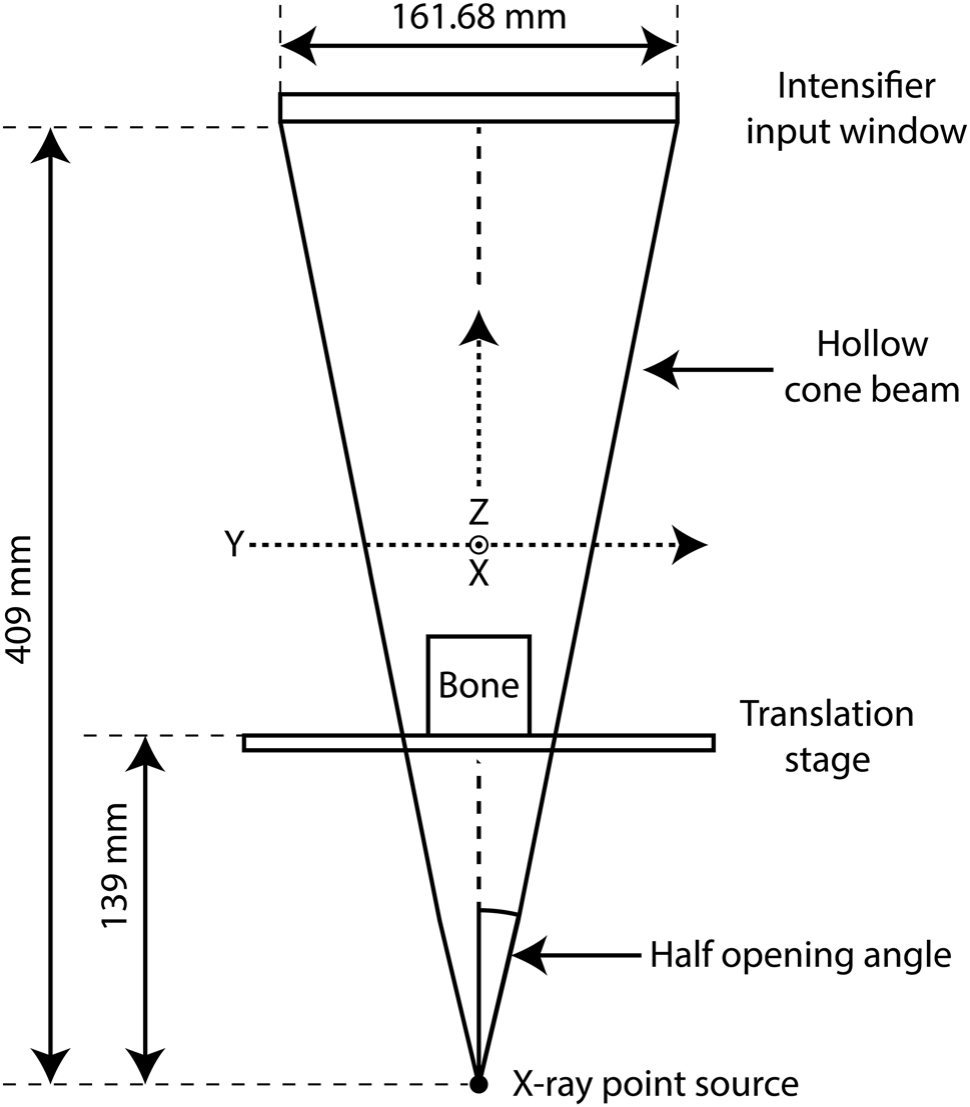
Instrument configuration. The bone sample sits on a translation stage, which enables a two axis x,y raster scan relative to the hollow beam. This arrangement is equivalent geometrically to raster scanning the beam over a stationary bone sample.

An FCG raster scan was performed similarly to that described previously^20,21,27^. A Hamamatsu L1261-07 microfocus X-ray source was used with an accelerating voltage and current of 150 kV and 67 µA, respectively. Conical projections were collected on a Siemens HIDEQ 33-4 ISX image intensifier with a CsI phosphor input screen (~ 160 mm diameter useful entrance field). The intensifier is optically coupled to a Videomed-XR2 GigE camera (1024 × 1024). The distance between the detector surface and the X-ray point source was fixed at 409 mm, with the bone sample and translation stage fixed 139 mm from the X-ray point source, as shown in Fig. 1.

The rectilinear raster scan was performed using 501 × 501 points with a 0.3 mm step size giving a 150 mm × 150 mm field of view for the digital tomosynthesis. A series of 18 concentric conical shell projections were measured at each scan step, with the mean annular ring radii linearly spaced from *r* = 7.89 mm to 75 mm, giving each a mean half opening from 1.11° to 10.39°, according to *tan*^-1^(*r*/*z*) where *z* = 409 mm.

A digital tomogram or sectional image was reconstructed from the ring projections using sparse interleaved sampling with 4× upscaling described in detail elsewhere^27^. Applying this method produced an image stack with 75 µm pixels in both *x* and *y* axes, with a slice thickness of 1 mm in *z*.

### Registration

**Figure 2:**
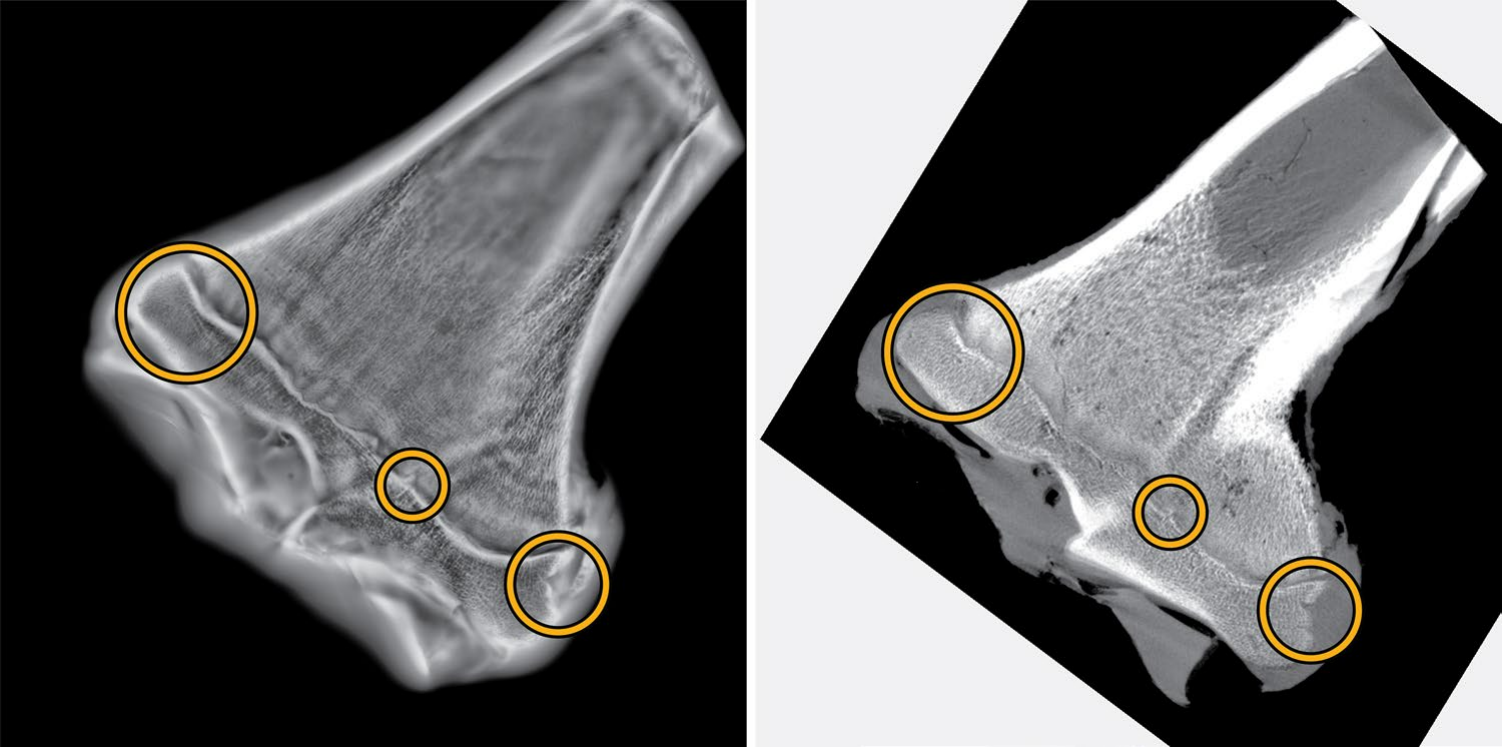
Landmarks used for registration of FCG (left) and µCT (right) slices.

To enable comparison of µCT and FCG images, a single slice of the FCG image stack was chosen for registration and three unique features were chosen within this slice (shown in Fig. 2). The µCT volume was rotated about each of its three orthogonal axes until the features chosen in the FCG slice were all in focus in one plane of the µCT volume. The uniqueness of the section was verified by examination of the entirety of both the FCG and µCT volumes. The accuracy of the relative in-plane registration was confirmed by comparative one-to-one coordinate analysis. The relative axial separations between the features were all found to be in good agreement, and within the limits of the predicted spatial resolution. Thus, the chosen features acted as surrogate fiducial markers of a unique plane within the experimental limits. The sectional image arising from this plane was used for all subsequent analysis.

Dissimilar *z*-axes resolution or section thickness produces an increase in FCG sectional image depth containing relatively more in-plane trabeculae.

To enable like-for-like comparison of µCT and FCG images, the FCG slice was downsampled to the same pixel size as the µCT image (125 µm) using the Matlab^28^ function *imresize* with bicubic interpolation. All further processing and analysis will be carried out on both the original FCG and downsampled FCG images.

### Feature exaggeration and artefact suppression

Blur arising from out-of-plane structures is a well-known difficulty when using shift-and-add reconstruction for tomosynthesis^29^ and is readily apparent within the FCG images seen here. When thresholding is attempted (either adaptive or global) poor results are seen where many trabeculae were not present, while large areas of cartilage and blur from out-of-focus planes are represented in the foreground (Fig. S1 in Supplemental Information).

While there are several deblurring algorithms and alternative reconstruction algorithms used within clinical tomosynthesis applications previously^29^, to combat these artefacts within this research a morphological top-hat filter was applied to reduce uneven background intensity. Top-hat (or white top-hat) transforms have been used in the past for clinical imaging purposes, for example for whole body sintigraphy^30^ and for examination of pathological calcifications^31,32^. To ensure consistent processing, both FCG and µCT images underwent the same processing procedure. All processing steps were carried out in Matlab^28^ and are given below:

- A disk structuring element was created with either *r* = 5 pixels (625µm) for µCT and downsampled FCG or *r* = 9 pixels (675µm) for FCG
- *imtophat* was used to morphologically open the image (erosion followed by dilation using the specified structuring element) then subtract the opened image from the original image
- *imadjust* was used to adjust image intensity values, saturating the upper 1% and lower 1% of the image.

The structuring element size was chosen to be larger than the features being targeted (trabecular thickness and separation). In this case, trabecular thickness in bovine trabecular bone is commonly reported between 110 and 600 µm, while trabecular separation is between 60 and 600 µm^33–37^. It should be noted though that most studies observe the femoral head or proximal tibia rather than the distal femur, and TbTh and TbSp have been seen to vary significantly based on the skeletal site and model used for calculation^33^.

**Figure 3:**
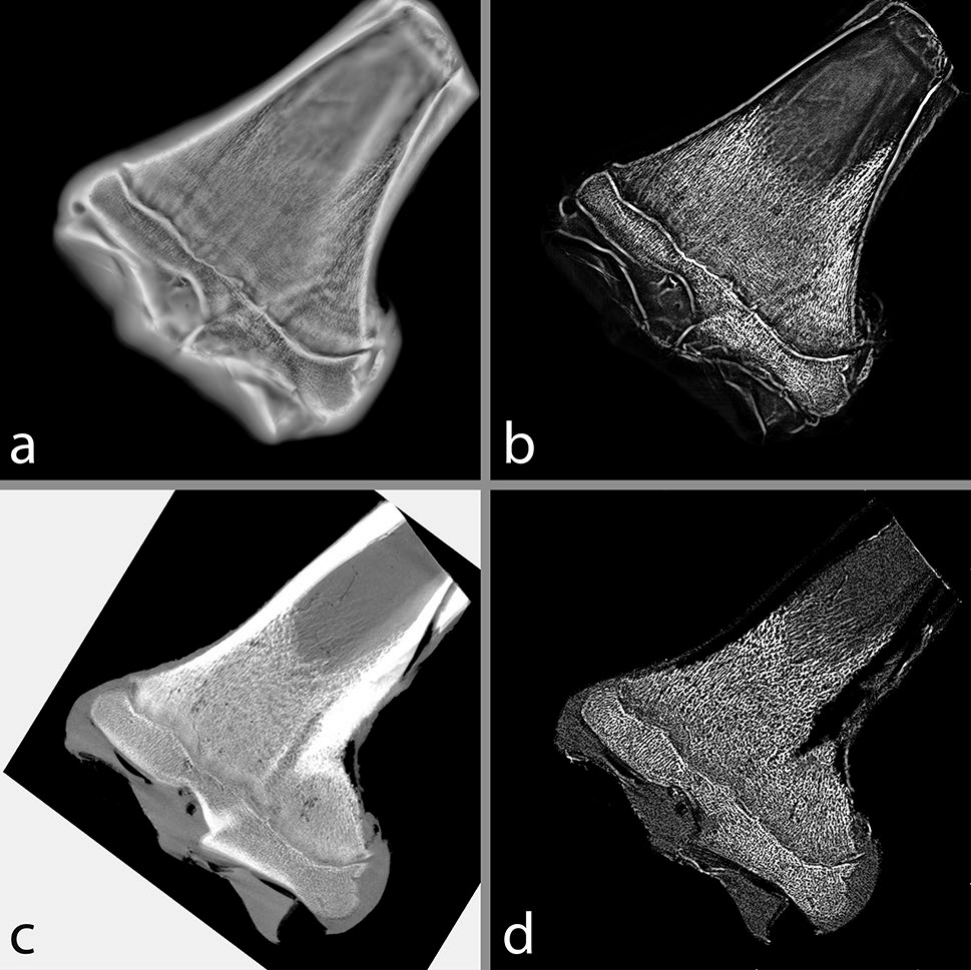
(**a**) initial and (**b**) processed FCG image, (**c**) initial and (**d**) processed µCT image.

The initial FCG image and processed FCG image are shown in Fig. 3a, b, respectively, while the initial µCT image and processed µCT image are shown in Fig. 3c, d, respectively. Initial images were not used for any further analysis, and only processed images will be used from this point forward. Within the images, it should be noted that most cortical bone ‘fades’ with the top-hat transform, as it is significantly larger than the structuring element used. This illustrates the importance in the choice of structuring element, particularly the radius (size).

### Binarization

**Figure 4:**
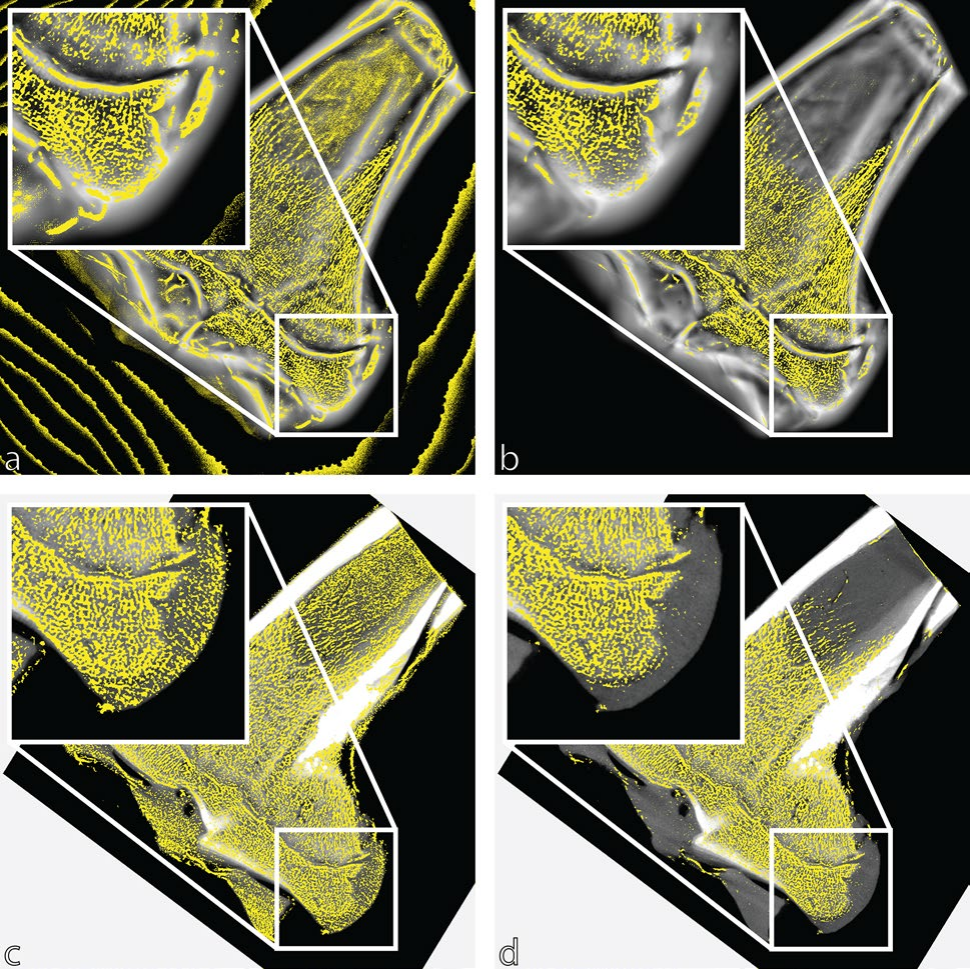
Binarized images for (**a**) adaptive thresholding of the FCG image, (**b**) global thresholding of the FCG image, (**c**) adaptive thresholding of the µCT image and (**d**) global thresholding of the µCT image.

Two binarization methods were used, adaptive and global, for each of the three images (µCT, FCG and downsampled FCG). Adaptive thresholding was applied using Bradley’s method^38^ with a neighbourhood size of either 151 × 151 pixels (µCT and downsampled FCG) or 251 × 251 pixels (FCG); the sensitivity value *s* is user specified based on visual inspection. For global thresholding, the threshold value *t* is specified by the user based on visual inspection. Figure 4 shows both adaptive and global binarizations for µCT and FCG images. An inset image shows a section of particular interest (due to the clearly visible landmarks) in greater detail. An apparent artifact can be seen in Fig. 4a, due to the top-hat transformation operating on a noisy background.

### Morphometric measurement

**Figure 5:**
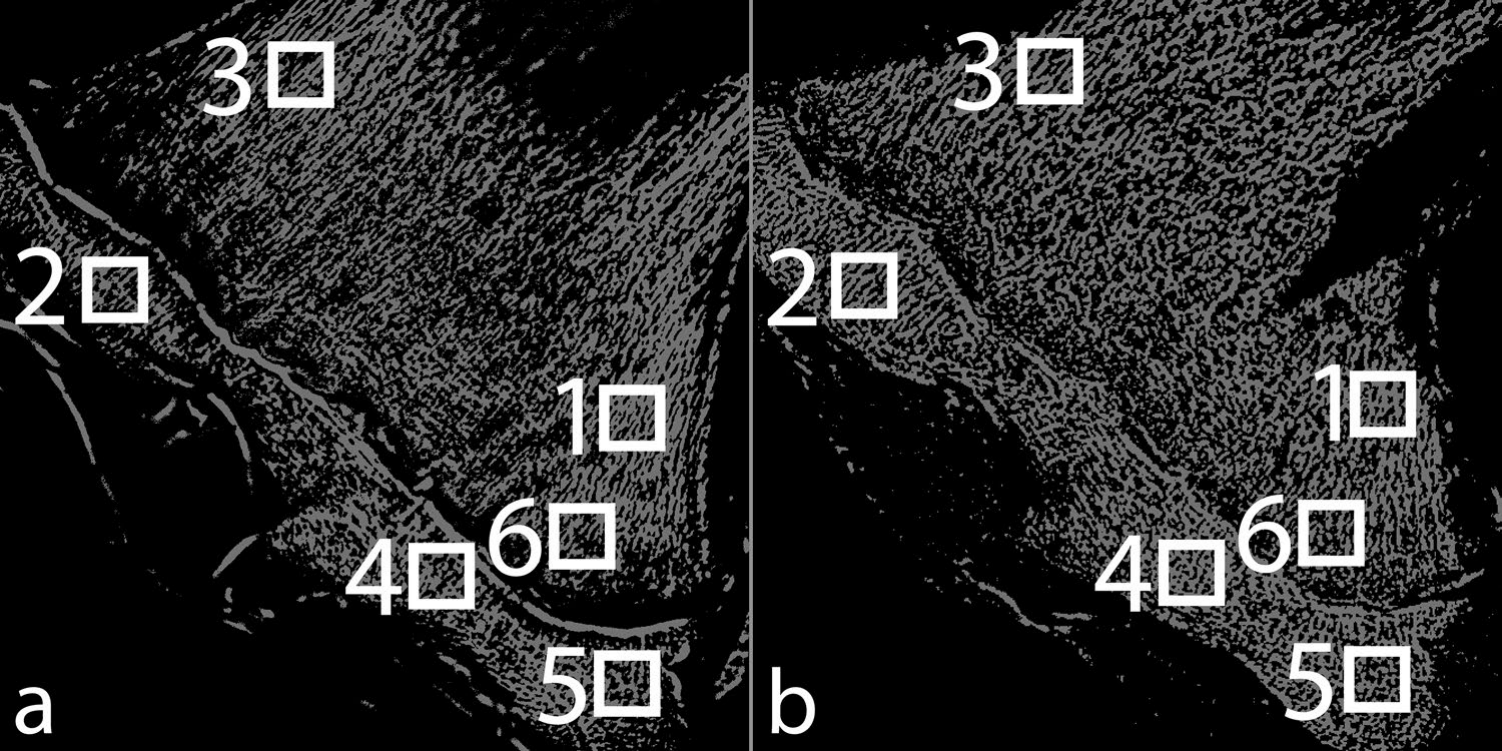
Selected regions of interest in the global binarization of (**a**) the FCG image and (**b**) the µCT image.

To interrogate accuracy of microarchitectural morphometrics, six regions of interest (ROIs) were selected in areas where trabecular bone was clearly visible for both global and adaptive binarizations of µCT and FCG images. Each region of interest was set as either 50 × 50 pixels (for µCT & downsampled FCG) or 83 × 83 (for FCG), seen in Fig. 5a, b for FCG and µCT, respectively.

**Figure 6:**
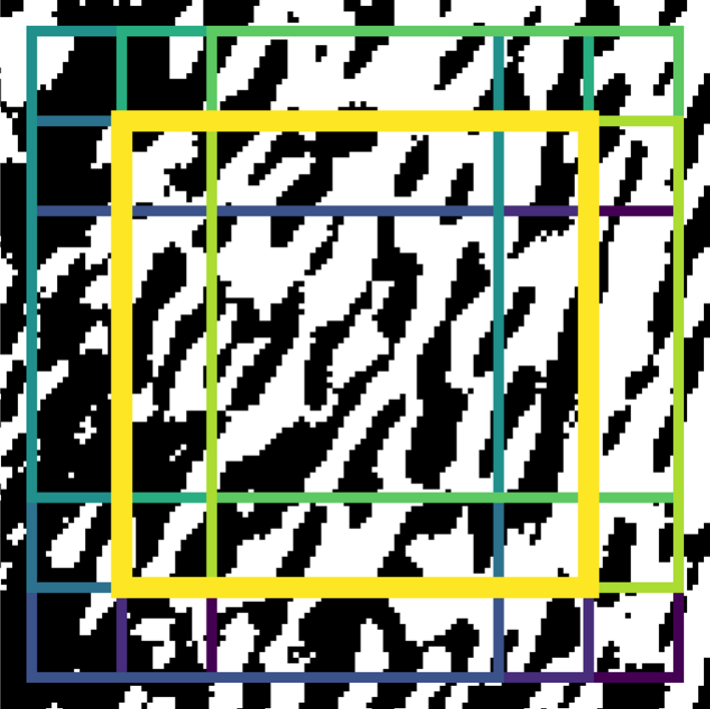
Example of the position of the nine sections taken from the adaptive binarization of the FCG image used which were analysed to produce data for region of interest one.

To test repeatability, each ROI was shifted to eight positions around the selected ROI (± 20% of ROI size), to produce a total of nine images which contributed to the morphometric measurement for each ROI (illustrated in Fig. 6). Mean and standard deviation was calculated for each set of nine images.

### Statistical analysis

Several morphometric parameters were determined using BoneJ^39,40^ in Fiji^41^: bone area/total area (BA/TA), fractal dimension (FD), trabecular thickness (TbTh) and trabecular separation (TbSp). Calculation of both TbTh and TbSp is not dependent upon either a plate or rod model but fits maximal circles to the structures in a model-independent method^42^. For each ROI in each of the six image types (both global and adaptive binarizations of µCT, FCG and downsampled FCG), each parameter measured was tested for normality using either a Shapiro–Wilk or a Shapiro-Francia test, depending on the kurtosis of the sample^43^. To determine significant differences between image type, if both samples being compared are normal, a two-sample T-test is performed assuming unequal variances; if one or both samples being compared are non-normal a Wilcoxon rank sum test was performed. In both cases, significance was set at *p* = 0.05.

## Results

### Qualitative comparison

**Figure 7:**
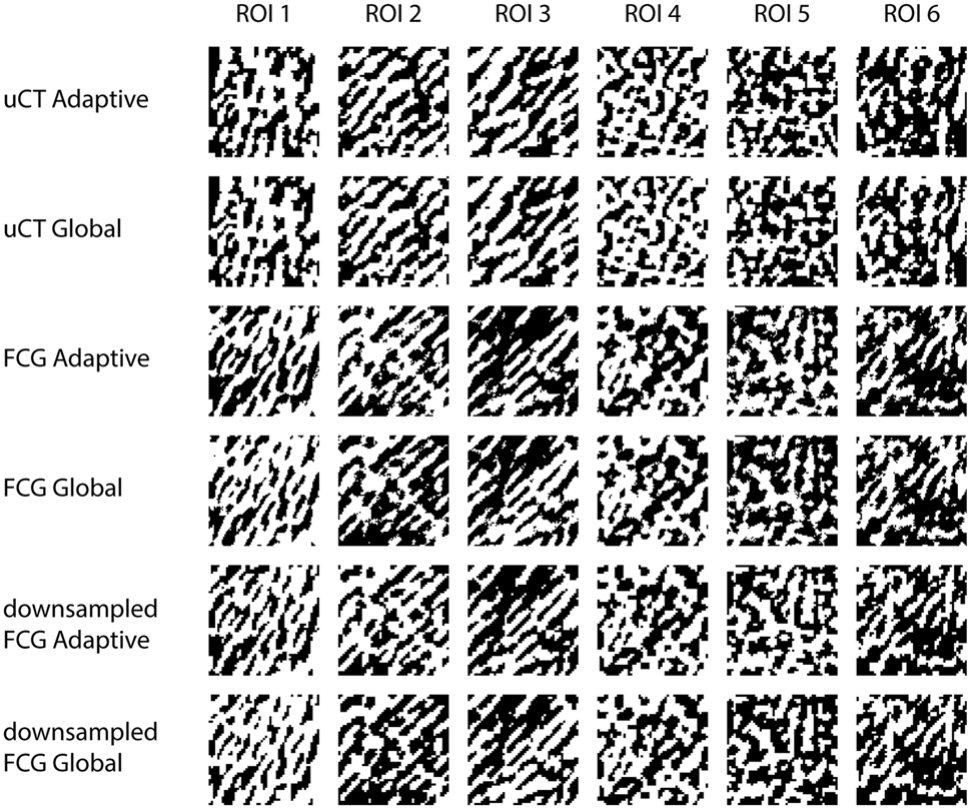
Regions of interest for each binarization method of each imaging technique.

Images from each ROI were compared for each imaging modality and each binarization method, shown in Fig. 7. While the difference in *z* resolution between the FCG image stack and µCT volume complicates the precise registration of individual trabeculae, consistent patterns within the trabecular structure can be observed. Implications of adaptive and global thresholding are considered further in the discussion.

### Quantitative comparison

**Figure 8:**
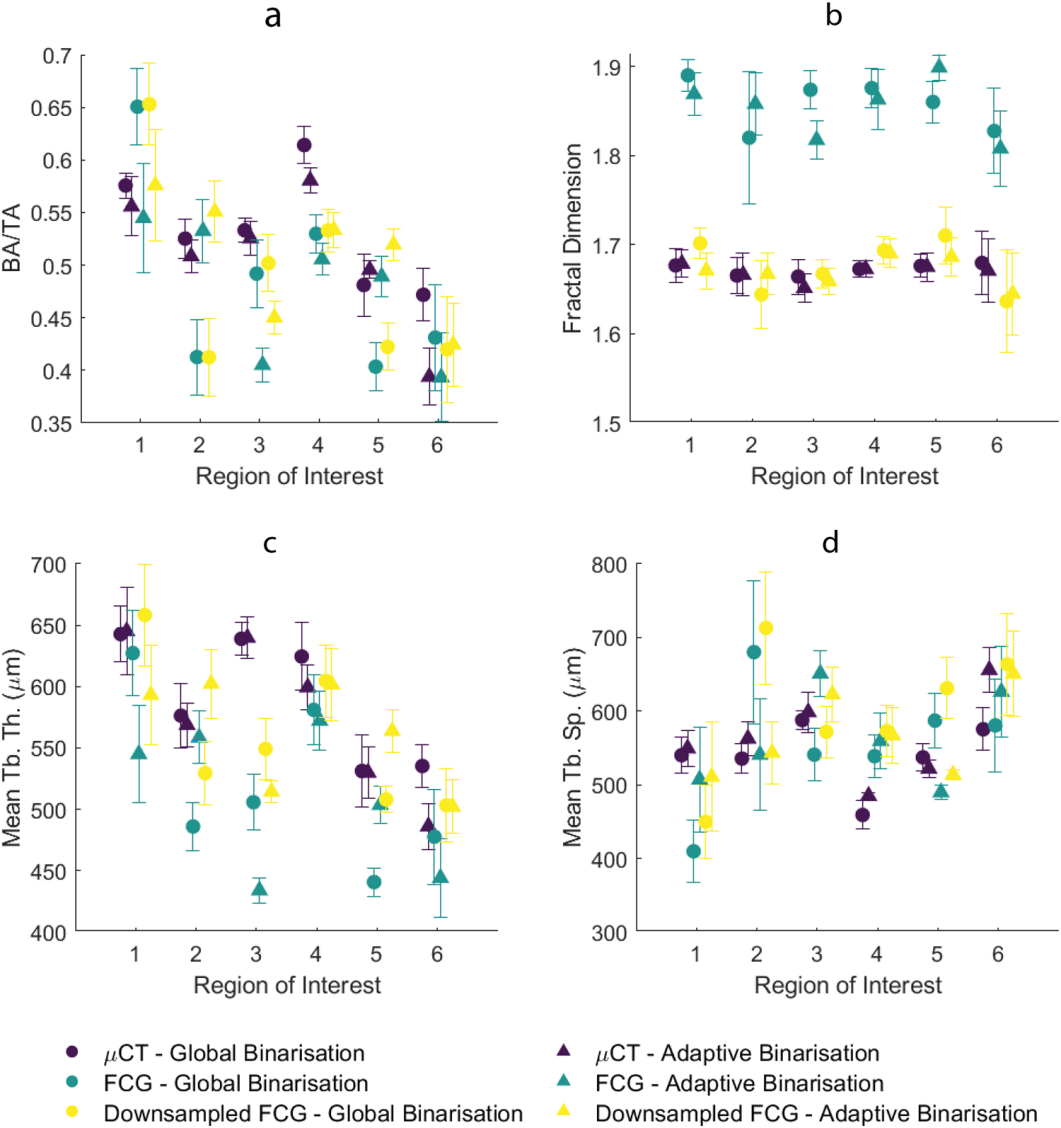
Morphometric parameters: (**a**) BA/TA, (**b**) fractal dimension, (**c**) mean TbTh and (**d**) mean TbSp. Error bars represent standard deviation for nine shifted images within each ROI.

Microarchitectural morphometrics were compared for both global and adaptive binarizations for each imaging modality. Results for all morphometric characteristics are given in Fig. 8, and results of all significance tests are given in Fig. S2 in Supplemental Information.

BA/TA for each ROI is shown in Fig. 8a. BA/TA values ranged from ~ 0.40 to ~ 0.65, varying with location, imaging method and binarization method. Several outliers are immediately noted, most often when the global binarization was used. Most ROIs give few significant differences between global and adaptive binarization of µCT images and taking the adaptive binarization of µCT as ground truth it is apparent most FCG and downsampled FCG images follow general trends. Perhaps most notable is the lack of significant differences between the adaptive binarization of FCG and µCT (p < 0.05 for only two of the six ROIs); this demonstrates the ability of FCG to measure BA/TA accurately compared to ‘ground truth’ µCT.

Fractal dimension (FD) shows much larger discrepancies between images with different pixel sizes (Fig. 8b). FD for FCG images is significantly different from all µCT and downsampled FCG images. In contrast, very few significant differences are seen between downsampled FCG and µCT images, particularly when an adaptive binarization is used (p < 0.05 for only ROI 4 when adaptive downsampled FCG is compared to both adaptive and global µCT). It is also of note that few significant differences are present when any adaptive binarization is compared to the global binarization from the same imaging mode (e.g. µCT, FCG or downsampled FCG).

When considering mean trabecular thickness (Fig. 8c), general trends can be observed for most ROIs. Similarly, mean trabecular separation (Fig. 8d) shows general agreement, particularly for adaptive binarizations. Both TbTh and TbSp show less of a pattern when significant differences between image types are examined. Of note is the different binarization techniques for each imaging modality, where at least two to four ROIs are significantly different (p < 0.05) for each. This result indicates that binarization method is very important for TbTh and TbSp measurement, particularly for FCG.

## Discussion

Thresholding has been seen to affect morphometric measurements in previous studies^44,45^, and is also apparent here. Differences resulting from adaptive or global thresholding were apparent in some parameters (BA/TA, TbTh and TbSp) while they did not affect others (such as FD). Also, some image types and binarizations are clear outliers (for example, global binarizations of both original FCG and downsampled FCG for BA/TA of ROI 2 and ROI 5), and often explained by the visual inspection of the ROI; for global binarizations of FCG at ROI 2 a large void is present near the lower part of the region, most likely due to the drastic change in background intensity in the original image, resulting in a relatively rapid intensity change of the trabeculae images after processing. This can also be seen in Fig. 4a, b insets, which show the area around ROI 5. Similarly a void can be seen for both global and adaptive binarizations at ROI 3, potentially explaining the differences for ROI 3 across imaging modalities.

This disagreement is perhaps demonstrated better in ROI 2, while the trabeculae near the growth plate are visually apparent in processed FCG images, the difference in intensity clearly introduces issues for global binarization. Close inspection of areas such as ROI 2 and ROI 5 with a high background intensity (and thus a potentially lower intensity of trabeculae after the top-hat transform) shows that while adaptive binarization technique accommodates this change in intensity better than the global binarization technique, it still does not ideally threshold. There is potential if the size of the neighbourhood used in the adaptive binarization is reduced, that this may allow more accurate thresholding of trabeculae network images with large changes in intensity, but it would potentially include more noise elsewhere in the image. This can be seen already for adaptive binarizations of both µCT and FCG images in Fig. 4a, c, where noise within the shaft of the femur has been discerned by the adaptive threshold due to the lack of intense features in the neighbourhood.

It is readily apparent that both global and adaptive binarizations can either over- or under-estimate trabeculae throughout the thresholding process if the thresholded region is sufficiently large. Choice of thresholding technique has been the focus of several studies in the past and has shown that different thresholding techniques affect different parameters to different degrees, as much as ~ 25%^44^, and parameters can also be affected by the observer^46^. Thresholding remains one of the most important considerations for the microarchitectural characterisation of trabecular bone.

Several previous studies have compared morphometrics from µCT to other imaging modalities, including cone beam CT (CBCT), multislice CT and MRI. In most cases, comparison was simply by correlation of parameters for each of the two imaging modalities^47–50^. Van Dessel et al*.* and Klintström et al*.* showed many parameters were significantly different from those measured by µCT, perhaps most importantly those parameters often used in bone disease research (e.g., BV/TV, TbTh, FD). Several studies reported morphometric values from either CBCT or MRI that were significantly different to corresponding µCT values, and consequently, both studies relied on the correlation of parameters between imaging techniques rather than comparison of absolute values^47,48,51^.

In this study, BA/TA values are consistent with literature values for the bovine femur and tibia, as are mean TbTh and TbSp values^33,36,37^. A previous study reported highly variable FD for different regions of bovine rib^52^ but FD observed within this study is consistent with those seen in both human and non-human studies previously^8,11,52^. Treating morphometrics measured by the µCT as a ground truth, it is apparent that FCG can precisely determine BA/TA and FD in most cases. Calculated FD has been seen to vary with different box size^53^ and different signal to noise ratios^54^, which may explain the different values seen between different pixel sizes (e.g., FCG versus downsampled FCG and µCT).

It can be seen that FCG can accurately measure FD, for an equivalent pixel size. Additionally, while FCG (particularly downsampled) accurately measures TbSp compared to µCT, it does not have the same accuracy measuring TbTh. This is potentially due to the circle fitting method imageJ uses, and potentially convoluted further by the relatively large step in *z* (1 mm).

While clearly the ability of FCG to image all microarchitectural characteristics accurately is affected by the decreased resolution available in the *z* axis, the potential for future exploitation of the geometry is profound. Simultaneous absorption and diffraction imaging using FCG has tremendous implications for the future of fracture prediction once both structural and physicochemical information is available.

A potential limitation of this study is the resolution of the µCT images; if a smaller voxel size could be achieved (i.e. by use of a smaller sample), morphometric results may be more precise. Though all efforts have been made to allow easy comparison with other imaging techniques used for morphometric analysis, some limitations are present. The presence of out-of-plane objects within the FCG image preclude this analysis from including traditional parameters commonly used in the clinical sphere such as TMD and _v_BMD. Microarchitectural characteristics measured are also limited to features within the 2D images investigated, and in the future a better average may be achieved by use of the 3D volume. This study has made every effort to follow guidelines accepted by the community for measurement of microarchitectural characteristics^45^.

## Conclusions

This study has shown that tomosynthesis using FCG presents an accurate measurement of microarchitectural morphometric parameters. FCG tomography allows for collection of both absorption and diffraction simultaneously^55^. The combination of absorption and diffraction FCG would provide the simultaneous interrogation of both morphometric and physicochemical bone quality via a single scan using the same hollow beam. Thus, given that such quality measures are known to be significantly altered with bone disease^9,56^, a combined absorption/diffraction probe promises significant clinical diagnostic utility.

### Supplementary Information

Supplementary Information 1.

Supplementary Figures.

Supplementary Information 2.

## Data Availability

Data supporting this study are included within the article and/or supplementary materials.

## Abbreviations

µCT: Micro-computed tomography
FCG: Focal construct geometry
ROI: Region of interest
BA/TA: Bone area/total area
FD: Fractal dimension
TbTh: Trabecular thickness
TbSp: Trabecular separation
DXA: Dual energy X-ray absorptiometry
BMD: Bone mineral density
HR-pQCT: High resolution peripheral quantitative CT
CBCT: Cone beam CT
MRI: Magnetic resonance imaging
XRD: X-ray diffraction
MSCT: Multislice CT
