# Supplementary figures and images for: Conical shell X-ray beam tomosynthesis and micro-computed tomography for microarchitectural characterisation
Source: Sci Rep. 2023 Dec 6;13:21480. doi: 10.1038/s41598-023-48851-6 (PMC10700317; doi:10.1038/s41598-023-48851-6)

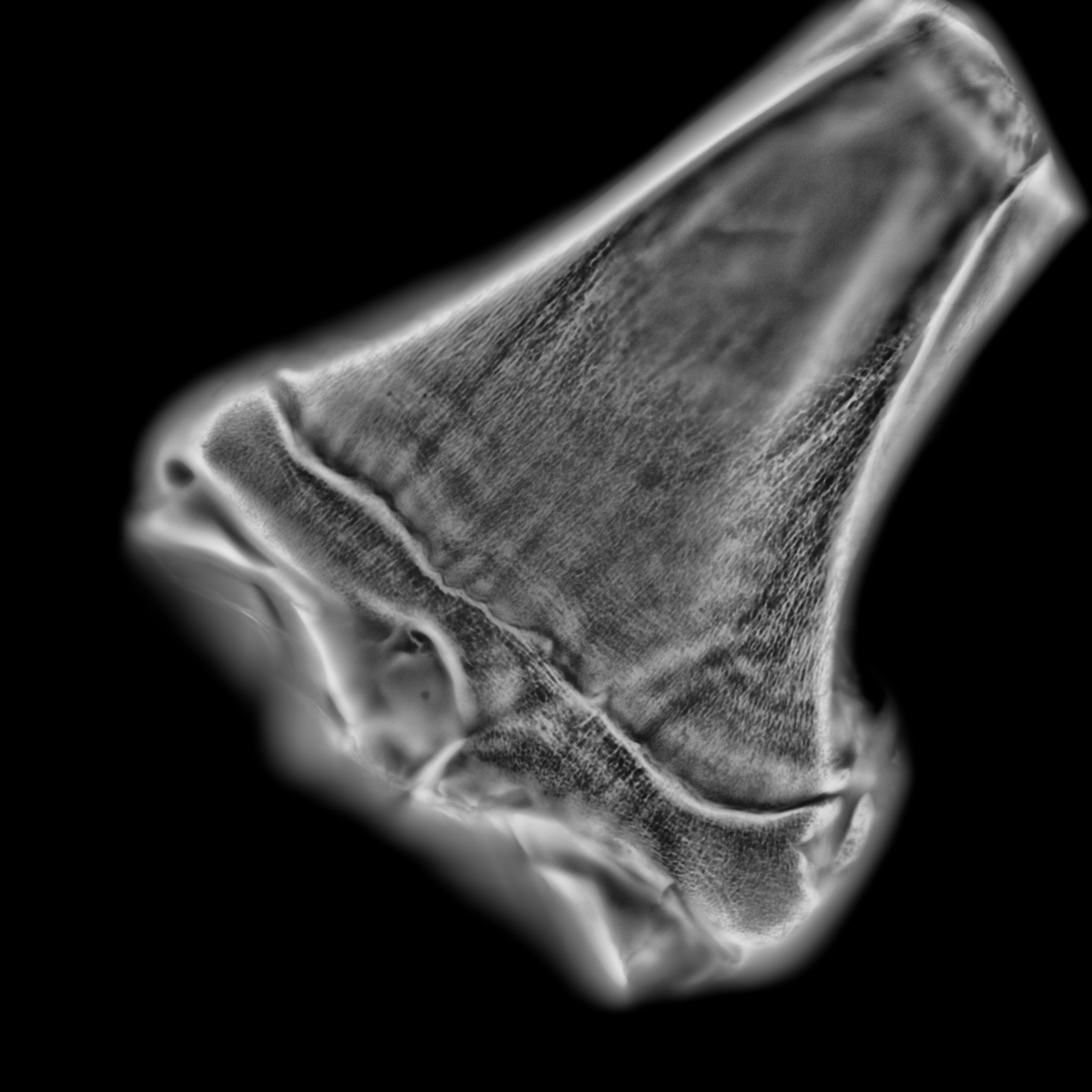

Supplement: Supplementary file 1 — Supplementary Information 1. [file 41598_2023_48851_MOESM1_ESM.png]

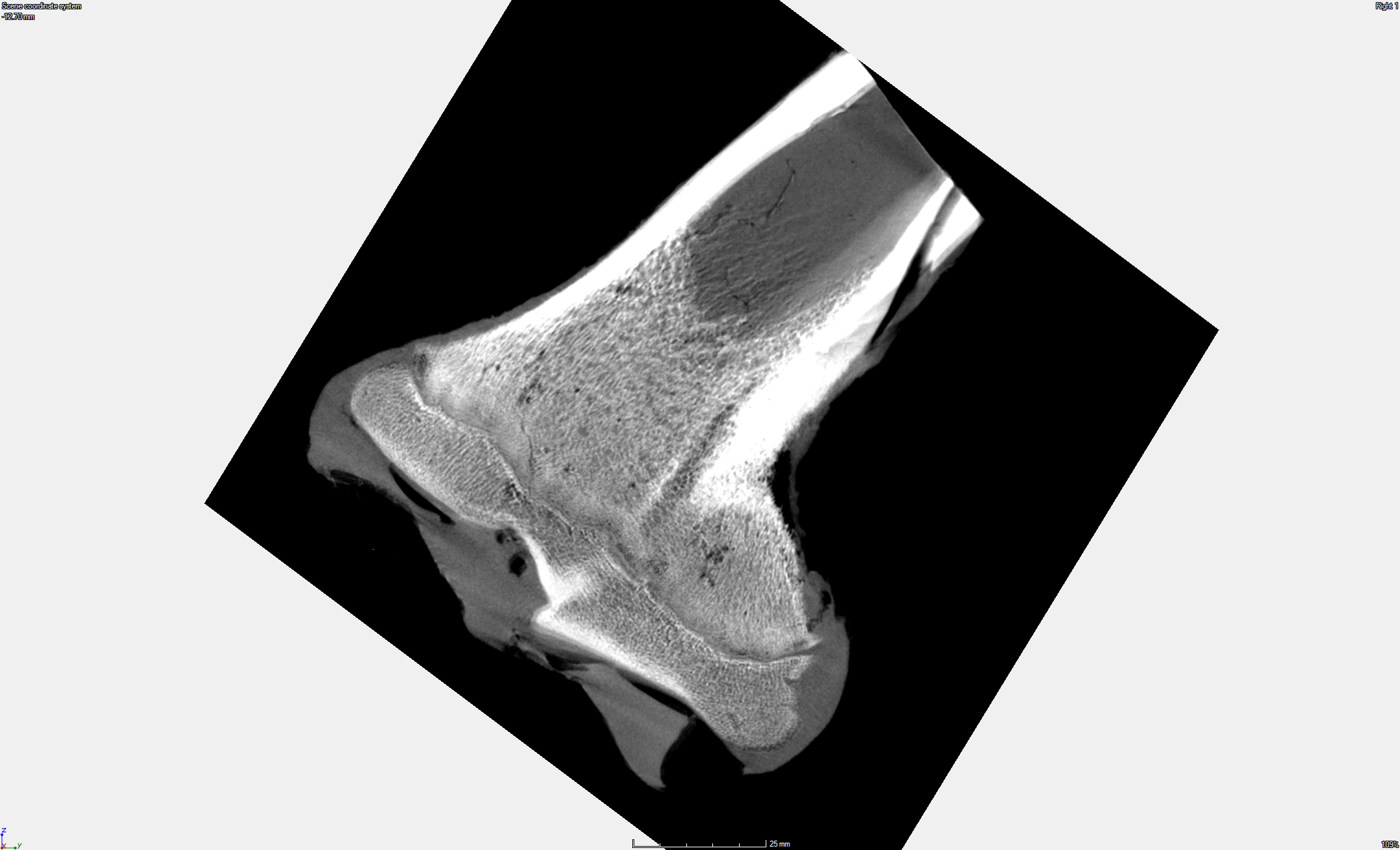

Supplement: Supplementary file 3 — Supplementary Information 2. [file 41598_2023_48851_MOESM3_ESM.tif]
