# Supplementary material for: Conical shell X-ray beam tomosynthesis and micro-computed tomography for microarchitectural characterisation
Source: Sci Rep. 2023 Dec 6;13:21480. doi: 10.1038/s41598-023-48851-6 (PMC10700317; doi:10.1038/s41598-023-48851-6)
Supplement: Supplementary file 2 — Supplementary Figures. [file 41598_2023_48851_MOESM2_ESM.pdf]

Supplementary Information

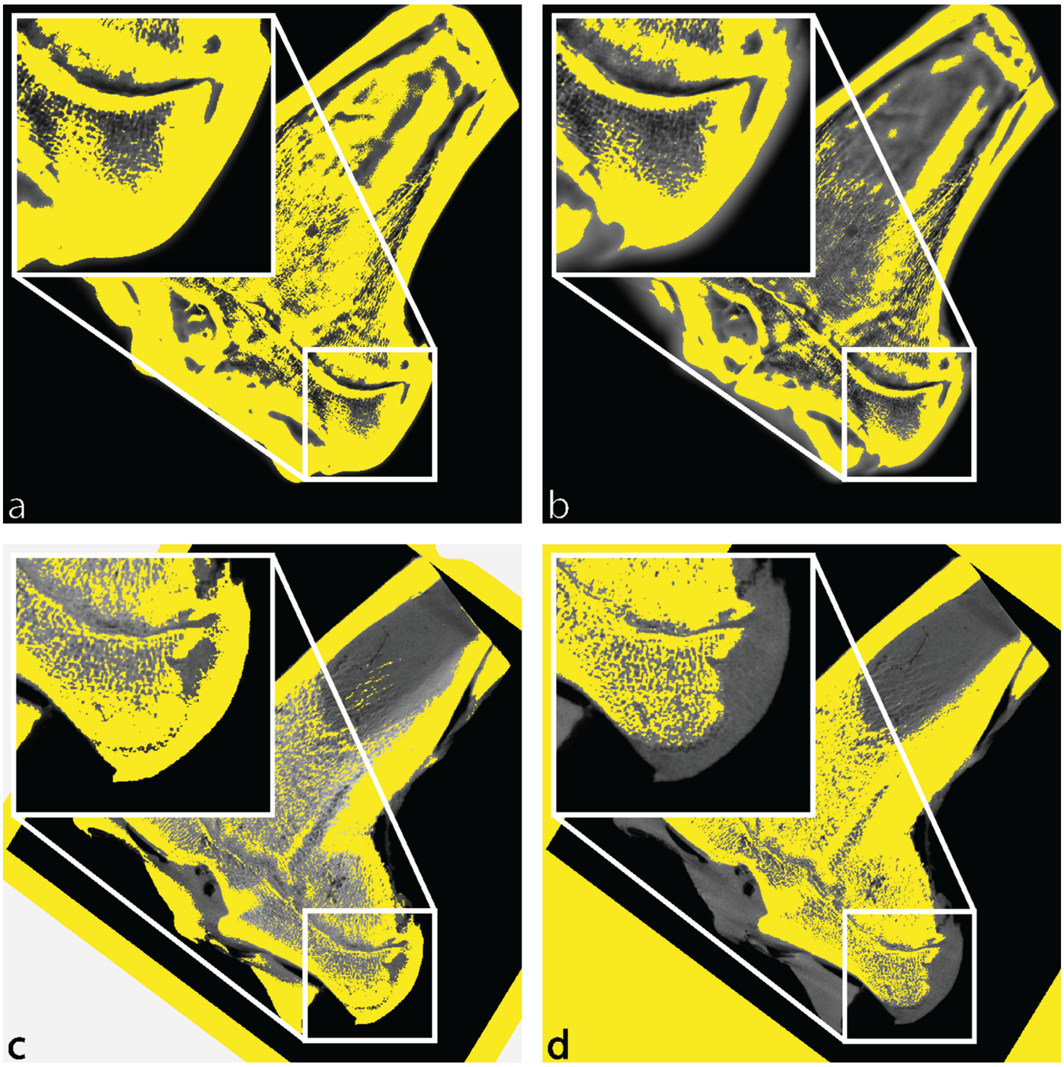

Figure S1: Initial thresholding attempts for FCG using a) adaptive binarization and b) global binarization; µCT using c) adaptive binarization and d) global binarization.

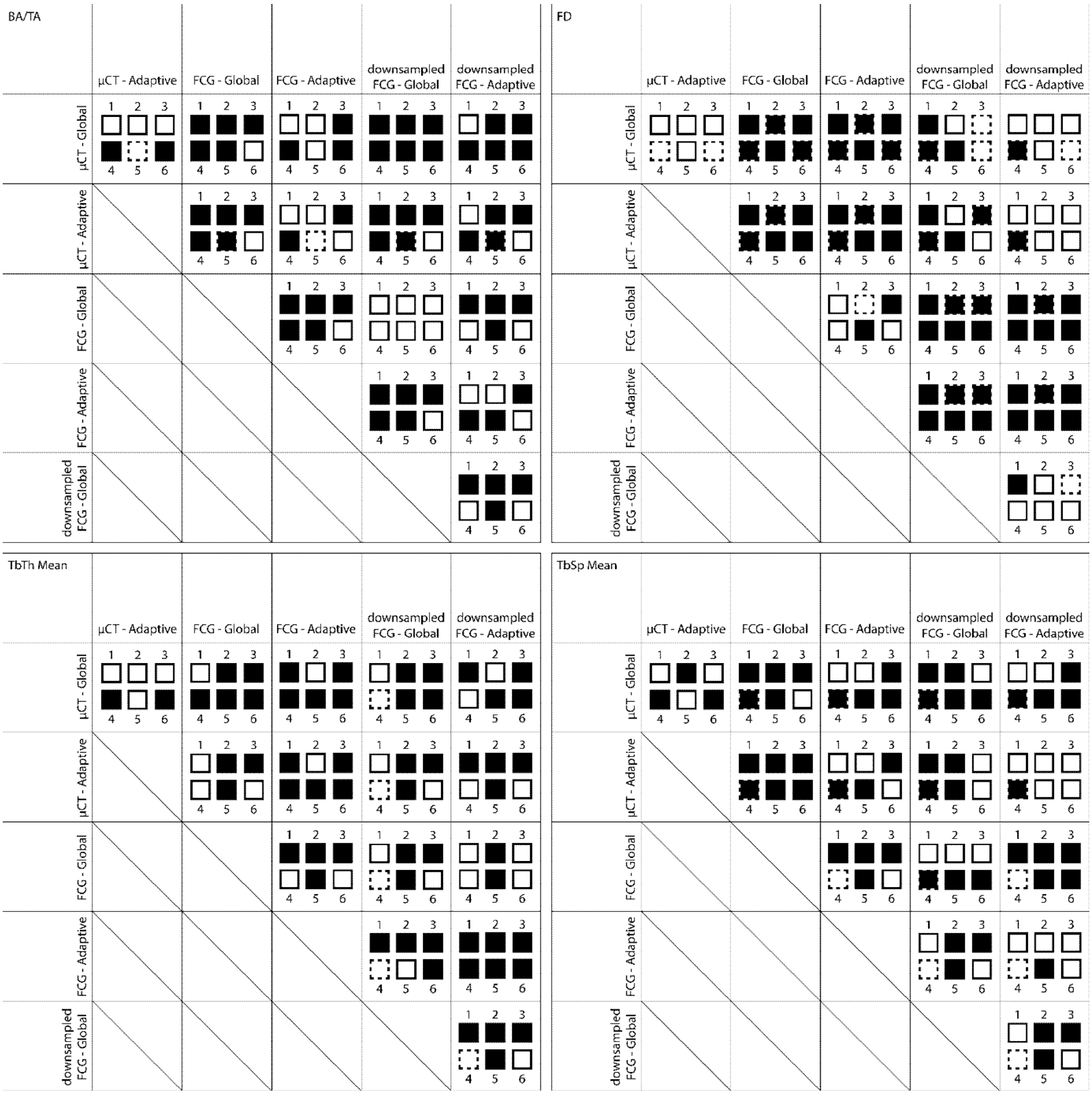

Figure S2: Results of statistical tests between each region of interest (ROI) for each image type, e.g. ROI 1 in the adaptively binarized µCT image was compared to ROI 1 in the globally binarized µCT image, ROI 2 in the adaptively binarized µCT image was compared to ROI 2 in the globally binarized µCT image and so on. Solid outlined boxes indicate where a two-sample T-test was performed, assuming unequal variance. Dashed outlined boxes indicate where a Wilcoxon rank sum test was performed (where one or more of the datasets were determined non-normal). A filled box represents where a significant difference ($p < 0.05$) was observed.
